# Comparison of clinical outcomes over time of inpatients with healthcare-associated or community-acquired coronavirus disease 2019 (COVID-19): A multicenter, prospective cohort study

**DOI:** 10.1017/ice.2023.143

**Published:** 2024-01

**Authors:** Rebecca L. Grant, Julien Sauser, Andrew Atkinson, Stéphanie D’Incau, Niccolò Buetti, Marie-Céline Zanella, Stephan Harbarth, Jonas Marschall, Gaud Catho, for the CH-SUR Collaborative Network

**Affiliations:** 1 Infection Control Program and WHO Collaborating Centre, Geneva University Hospitals and Faculty of Medicine, Geneva, Switzerland; 2 Department of Infectious Diseases, Bern University Hospital, Inselspital, University of Bern, Bern, Switzerland; 3 Division of Infectious Diseases, Washington University School of Medicine, St. Louis, Missouri, United States; 4 Division of Infectious Diseases, Lucerne Cantonal Hospital, Lucerne, Switzerland; 5 Division of Infectious Diseases, Central Institute, Valais Hospital, Sion, Switzerland

## Abstract

**Objective:**

To compare clinical outcomes over time of inpatients with healthcare-associated coronavirus disease 2019 (HA-COVID-19) versus community-acquired COVID-19 (CA-COVID-19).

**Design:**

We conducted a multicenter, prospective observational cohort study of inpatients with COVID-19.

**Setting:**

The study was conducted across 16 acute-care hospitals in Switzerland.

**Participants and methods:**

We compared HA-COVID-19 cases, defined as patients with a positive severe acute respiratory coronavirus virus 2 (SARS-CoV-2) test > 5 days after hospital admission, with hospitalized CA-COVID-19 cases, defined as those who tested positive within 5 days of admission. The composite primary outcome was patient transfer to an intensive care unit (ICU) or an intermediate care unit (IMCU) and/or all-cause in-hospital mortality. We used cause-specific Cox regression and Fine-Gray regression to model the time to the composite clinical outcome, adjusting for confounders and accounting for the competing event of discharge from hospital. We compared our results to those from a conventional approach using an adjusted logistic regression model where time-varying effects and competitive risk were ignored.

**Results:**

Between February 19, 2020, and December 31, 2020, we included 1,337 HA-COVID-19 cases and 9,068 CA-COVID-19 cases. HA-COVID-19 patients were significantly older: median, 80 (interquartile range [IQR], 71–87) versus median 70 (IQR, 57–80) (*P* < .001). A greater proportion of HA-COVID-19 patients had a Charlson comorbidity index ≥ 5 (79% vs 55%; *P* < .001) than did CA-COVID-19 patients. In time-varying analyses, between day 0 and 8, HA-COVID-19 cases had a decreased risk of death or ICU or IMCU transfer compared to CA-COVID-19 cases (cause-specific hazard ratio [csHR], 0.43; 95% confidence interval [CI], 0.33–0.56). In contrast, from day 8 to 30, HA-COVID-19 cases had an increased risk of death or ICU or IMCU transfer (csHR, 1.49; 95% CI, 1.20–1.85), with no significant effect on the rate of discharge (csHR, 0.83; 95% CI, 0.61–1.14). In the conventional logistic regression model, HA-COVID-19 was protective against transfer to an ICU or IMCU and/or all-cause in-hospital mortality (adjusted odds ratio [aOR], 0.79, 95% CI, 0.67–0.93).

**Conclusions:**

The risk of adverse clinical outcomes for HA-COVID-19 cases increased substantially over time in hospital and exceeded that for CA-COVID-19. Using approaches that do not account for time-varying effects or competing events may not fully capture the true risk of HA-COVID-19 compared to CA-COVID-19.

The coronavirus disease 2019 (COVID-19) pandemic has placed an enormous burden on healthcare systems, both in the number of patients seeking care and the need for implementation of appropriate infection prevention and control measures to prevent healthcare transmission of severe acute respiratory coronavirus virus 2 (SARS-CoV-2). In terms of patient outcomes, major known risk factors for severe COVID-19 include older age, pregnancy, and the presence of comorbidities such as BMI ≥30 kg/m^2^, chronic lung disease, diabetes, chronic cardiovascular disease, or chronic renal disease, malignancy and immunosupression.^1,2^

Given the clinical characteristics and fragility of hospitalized patients, there is concern that healthcare-associated SARS-CoV-2 infection may result in worse clinical outcomes compared to community-acquired COVID-19 (CA-COVID-19). Indeed, outbreaks of COVID-19 in long-term care facilities and geriatric wards have repeatedly demonstrated high mortality rates among patients, particularly prior to the introduction of COVID-19 vaccines.^3–6^ However, there is limited evidence regarding whether hospital acquisition of COVID-19 is a determinant of severe clinical outcomes and/or in-hospital mortality. A recent meta-analysis of 21 studies reporting outcomes of nosocomial and CA-COVID-19^7^ included 8,251 hospital admissions across 8 countries from January 2020 to February 2021. The pooled relative risk of in-hospital mortality among nosocomial and CA-COVID-19 cases indicated that nosocomial COVID-19 cases had 1.3 times increased risk of mortality (95% confidence interval [CI], 1.005–1.683) and a similar risk of requiring critical care (relative risk [RR], 0.74; 95% CI, 0.50–1.08) compared to CA-COVID-19 cases.^7^ However, the definitions of nosocomial acquisition of SARS-CoV-2 between studies were heterogeneous, ranging from 2 to 14 days from admission to diagnosis of COVID-19. Moreover, the quality of evidence was assessed to be very low for most of the studies, with high risk of bias in representativeness, heterogeneity in the definitions of HA-COVID-19 cases and low comparability of control populations. Importantly, none of the studies investigated the variation in clinical outcomes over time, or the impact of length of hospital stay.

We assessed clinical outcomes of HA-COVID-19 cases compared to hospitalized CA-COVID-19 cases in Switzerland. Specifically, we assessed how the risk of adverse clinical outcomes may vary over time and how appropriate statistical analyses provide a more granular assessment regarding the true effect estimate of HA-COVID-19.

## Methods

### Study design and setting

The hospital-based surveillance of COVID-19 in Switzerland (CH-SUR) is a prospective cohort study of COVID-19 inpatients in 20 hospitals across Switzerland, including all of the major tertiary-care centers and Switzerland’s 5 university hospitals, which was initiated on February 19, 2020.^8,9^ We conducted a multicenter prospective observational cohort study across acute-care hospitals in Switzerland participating in CH-SUR between February 19, 2020, and December 31, 2020. Of the 20 hospitals involved in CH-SUR, the 16 that were actively participating and enrolling patients during the study period were included in this analysis. The participating hospitals included the 5 university hospitals in Switzerland, as well as 11 regional public or private hospitals.

### Inclusion in analysis

We included all consecutive COVID-19 inpatients admitted for at least 24 hours to 1 of the 16 participating CH-SUR hospitals between February 19, 2020, and December 31, 2020 (corresponding to the first and second pandemic waves in Switzerland). Patients eligible for inclusion in the analysis were aged ≥18 years and had laboratory confirmation of SARS-CoV-2 infection by reverse-transcription polymerase chain reaction (RT-PCR) obtained from any type of respiratory specimen.

Given that the median incubation period for SARS-CoV-2 was estimated to be ∼5 days in 2020,^10^ HA-COVID-19 cases were defined by a PCR-confirmed positive SARS-CoV-2 respiratory specimen, or by the onset of symptoms compatible with COVID-19 >5 days after hospital admission, whichever came first. Hospitalized CA-COVID-19 cases were considered those with a PCR-confirmed positive SARS-CoV-2 respiratory specimen or onset of signs and symptoms compatible with COVID-19 before or up to 5 days after hospital admission. This included patients who were admitted to one of the participating CH-SUR hospitals directly, as well as patients who may have been admitted in another health facility for <5 days before being transferred to one of the CH-SUR hospitals.

### Exclusion criteria

Patients <18 years of age, those whose date of symptom onset, date of hospitalization or date of specimen collection data did not allow for distinction of HA-COVID-19 from CA-COVID-19, or for whom outcome data were missing, were excluded from the analysis. Patients who reported previous COVID-19 (predating the current hospitalization) were also excluded from the analysis, as were patients who acquired SARS-CoV-2 while admitted to an intensive care unit (ICU) or an intermediate care unit (IMCU).

### Data collection

Investigators at each site used a standardized electronic case report form for data collection hosted by Research Electronic Data Capture Database (REDCap; Nashville, TN) that was based on a similar, previously validated tool for influenza.^8^ Investigators collecting data were study nurses or research assistants who had been trained in data collection. Data collected from each patient included demographic, clinical information at baseline and therapeutic and outcome information. Clinical data on comorbidities were summarized using the Charlson comorbidity index (CCI).

### Outcome measures

The primary outcome measures were patient transfer to an ICU or IMCU and/or all-cause in-hospital mortality, whichever occurred first.

### Statistical analyses

The demographic and clinical characteristics of HA-COVID-19 cases and CA-COVID-19 cases were compared using the Wilcoxon rank-sum test for continuous variables and Pearson’s χ^2^ test for categorical variables.

For HA-COVID-19 cases, hospital stay in days was measured from either ‘date of symptom onset’ or ‘date of first SARS-CoV-2 respiratory specimen,’ whichever occured first. For CA-COVID-19 cases, hospital stay in days was measured from the date of hospital admission, to avoid immortal time bias. Duration of hospital stay was measured until either patient transfer to ICU or IMCU, all-cause in-hospital death or discharge from hospital, whichever occurred first.

In addition, three different analyses were performed to evaluate the impact of HA-COVID-19 on clinical outcomes. First, the relative risk of in-hospital mortality, or patient transfer to an ICU or IMCU for HA-COVID-19 cases as compared to CA-COVID-19 cases was calculated as the proportion of HA-COVID-19 cases who were transferred to an ICU or IMCU or who died in the hospital, as compared to the proportion of CA-COVID-19 cases who were transferred to an ICU or IMCU or who died in the hospital.

Second, we used multivariable mixed-effects logistic regression analyses to evaluate the association of HA- versus CA-COVID-19 on the primary composite end point. Known or documented factors associated with adverse clinical COVID-19 outcomes were included in the multivariable logistic regression model. The primary composite end point was measured on day 30, and indicated whether a patient had either died or had been transferred to an ICU or IMCU. Hospital-specific random effects were used to account for clustering. Participating hospitals were pooled by location when there were several hospitals in a town or in a close geographic area.

Finally, we calculated the cumulative incidence of the primary composite end point using the Aalen-Johansen estimator. To avoid overestimation of the primary outcome for hazard-based models, we accounted for competing risks, defined as patient discharge alive from hospital.^10^ Time to the composite primary end point for HA-COVID-19 cases and CA-COVID-19 cases were then analyzed both as proportional cause-specific hazard ratios (csHRs) using multivariable Cox regression models and as subdistribution hazard ratios (sHRs) using the Fine-Gray proportional hazards model, considering clustering at the hospital level and with discharge from hospital as a competing event.^11,12^ Effect estimates were reported with an associated 95% confidence interval (95% CI). For these models, patients who were hospitalized for COVID-19 for >30 days were right censored at day 30. In addition, multiple imputation was used for missing covariate data assuming a missing-at-random mechanism. We assessed whether the proportional hazards assumption over the follow-up period was met, and where it was not met, follow-up time was partitioned.

All analyses were performed using R version 4.02 software (http://cran.r-project.org/). Two-tailed tests were performed, and *P* < .05 was considered statistically significant.

### Ethical considerations

The CH-SUR project was submitted to and approved by the Geneva Ethics Committee (CCER 2018-00577) and by the local ethics committee in each participating hospital through the Swiss Business Administration System for Ethics Committees (BASEC) submission system (no. 2020-00827).

## Results

Between February 19, 2020, and December 31, 2020, a total of 11,169 hospital stays for COVID-19 patients were entered into the CH-SUR database and were available for analysis. Among them, 764 patients were excluded for the following reasons: 267 were aged <18 years; 16 reported previous COVID-19; 311 were not able to be classified as HA-COVID-19 or CA-COVID-19 cases; and 170 had missing data for the primary outcome.

The remaining 10,405 patients were included in the analysis: 9,068 were classified as CA-COVID-19 cases and 1,337 were classified as HA-COVID-19 cases. Table 1 describes the baseline demographic and clinical characteristics of the study patients. Compared to CA-COVID-19 cases, HA-COVID-19 patients were significantly older: median, 80 years (IQR, 71–87) versus 70 years (IQR, 57–80) (*P* < .001). A greater proportion of HA-COVID-19 patients were female (48.9% vs 39.6%; *P* < .001) and were more likely to have a Charlson comorbidity index ≥5 (78.6% vs 55.0%; *P* < .001) (Table 1).

**Table 1.** Baseline Demographic and Clinical Characteristics of HA-COVID-19 and CA-COVID-19 Patients

Characteristic	HA-COVID-19 (N=1,337), No. (%)^a^	CA-COVID-19 (N=9,068), No. (%)^a^	*P* Value
Age, median y [IQR]	80 [71–87]	70.00 [57–80]	<.001
Sex, female	654 (48.9)	3591 (39.6)	<.001
BMI, median kg/m² [IQR]^b^	24.4 [20.9–28.1]	26.9 [23.8–30.9]	<.001
**Comorbidities^c^**			
Chronic respiratory disease	239/1,109 (21.4)	1,254/6,521 (19.2)	
Diabetes mellitus	307/1,108 (27.7)	1,995/6,530 (30.6)	
Chronic cardiovascular disease	615/1,103 (55.8)	2,582/6,524 (39.6)	
Chronic renal disease	377/1,106 (34.1)	1,433/6,516 (21.9)	
Current malignancy	248/1,102 (22.5)	857/6,519 (13.1)	
**Charlson comorbidity index**			<.001
1–2	57 (4.3)	1,514 (16.7)	
3–4	210 (15.7)	1,852 (20.4)	
≥5	1,051 (78.6)	4,986 (55.0)	
**Place of residence before hospitalization**			
Home	932 (69.7)	7,605 (83.9)	<.001
Other acute-care hospital	170 (12.7)	592 (6.5)	
Long-term care facility	151 (11.3)	477 (5.3)	
Rehabilitation center	37 (2.8)	43 (0.5)	
Other	39 (2.9)	162 (1.8)	
Missing	8 (0.6)	189 (2.1)	
**Participating hospital^d^**			
1	232 (17.4)	1,375 (15.2)	
2	229 (17.1)	1,258 (13.9)	
3	1 (0.1)	5 (0.1)	
4	2 (0.1)	75 (0.8)	
5	70 (5.2)	791 (8.7)	
6	459 (34.3)	1,761 (19.4)	
7	31 (2.3)	138 (1.5)	
8	5 (0.4)	142 (1.6)	
9	0 (0.0)	53 (0.6)	
10	124 (9.3)	1,415 (15.6)	
11	58 (4.3)	671 (7.4)	
12	6 (0.4)	68 (0.7)	
13	5 (0.4)	208 (2.3)	
14	0 (0.0)	1 (0.0)	
15	50 (3.7)	481 (5.3)	
16	65 (4.9)	626 (6.9)	
**Initial admission ward**			
General medicine	549 (41.1)	6,722 (74.1)	<.001
Geriatrics/Rehabilitation	404 (30.2)	437 (4.8)	
ICU or IMCU	68 (5.1)	875 (9.6)	
Surgery	193 (14.4)	222 (2.4)	
Emergency department	8 (0.6)	265 (2.9)	
Gynecology	4 (0.3)	86 (0.9)	
Psychiatry	46 (3.4)	41 (0.4)	
Other	57 (4.3)	258 (2.8)	
Missing	*8 (0.6)*	*162 (1.8)*	

Note. HA-COVID-19, healthcare-associated COVID-19; CA-COVID-19, community-acquired COVID-19; ICU, intensive care unit; IMCU, intermediate care unit.

a Units unless otherwise specified.

b Missing data for 2,372 patients (2,224 CA-COVID-19 cases and 148 HA-COVID-19 cases).

c Reporting of comorbidities was not mandatory and therefore omitted by some centers.

d The 16 hospitals were grouped in 11 regions or cities for the analysis.

### Primary outcomes

Table 2 provides an overview of the clinical outcomes for HA-COVID-19 and CA-COVID-19. The mortality rate among HA-COVID-19 cases was 18.3%, and 8.8% of HA-COVID-19 cases were transferred to an ICU or IMCU during the follow-up period. The mortality rate among CA-COVID-19 cases was 7.7%, and 21.8% of CA-COVID-19 were transferred to an ICU or IMCU during the follow-up period.

**Table 2.** Crude Clinical Outcomes for HA-COVID-19 and CA-COVID-19 Cases

Outcome	HA-COVID-19 (N=1,337), No. (%)	CA-COVID-19 (N=9,068), No. (%)	Total (N=10,405), No. (%)
Death	245 (18.3)	696 (7.7)	941 (9.0)
Transfer to an ICU or IMCU	118 (8.8)	1,979 (21.8)	2,097 (20.2)
Right censored at day 30	331 (24.8)	628 (6.9)	959 (9.2)
Discharge from hospital alive	643 (48.1)	5,765 (63.6)	6,408 (61.6)

Note. HA-COVID-19, healthcare-associated COVID-19; CA-COVID-19, community-acquired COVID-19; ICU, intensive care unit; IMCU, intermediate care unit.

The relative risk of the composite primary end point for HA-COVID-19 compared to CA-COVID-19 was 1.15 (95% CI, 1.07–1.23). In conventional, multivariable logistic regression analysis, HA-COVID-19 was identified as an independent protective factor for patient transfer to an ICU or IMCU or all-cause in-hospital mortality (aOR, 0.79; 95% CI, 0.67–0.93) (Table 3). Treatment with corticosteroids was an independent risk factor for adverse clinical outcomes (aOR, 2.39; 95% CI, 2.10–2.73); however, this likely reflects the use of these therapeutics in more severe patients.

**Table 3.** Multivariable Logistic Regression Model^a^ Predicting Patient Transfer to an ICU or ICMU or All-Cause In-Hospital Mortality for 10,405 Patients Included in the Analysis

Characteristic	aOR	95% CI	*P* Value
HA-COVID-19	0.79	0.67–0.93	.005
Sex, female	0.59	0.52–0.66	<.001
Age ≥65 y	1.26	1.10–1.45	.001
BMI ≥30 kg/m²	1.16	1.02–1.32	.02
Chronic cardiovascular disease	1.22	1.07–1.38	.002
Diabetes	1.21	1.07–1.37	.003
Chronic respiratory disease	1.31	1.14–1.50	<.001
Chronic renal disease	1.23	1.08–1.42	.003
Current malignancy	1.11	0.94–1.29	.20
Corticosteroid treatment for COVID-19	2.39	2.10–2.73	<.001

Note. aOR, adjusted odds ratio; CI, confidence interval; BMI, body mass index; HA-COVID-19, healthcare-associated COVID-19; CA-COVID-19, community-acquired COVID-19; ICU, intensive care unit; IMCU, intermediate care unit.

a Multivariate regression model adjusted for acquisition (community acquired or healthcare associated), sex, age (categorized as <65 and ≥65 years), BMI (categorized as < 30 or ≥30 kg/m²), chronic lung disease, diabetes, chronic cardiovascular disease, chronic renal disease, malignancy and administration of corticosteroid treatment for COVID-19.

**Figure 1.**
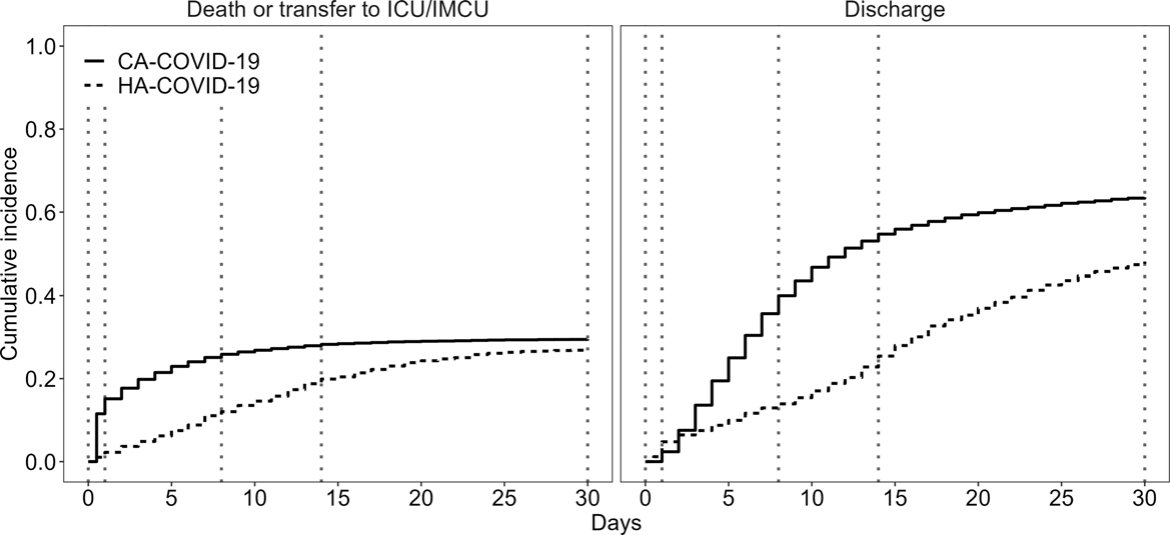
Cumulative incidence curves of patient transfer to an ICU or IMCU or all-cause in-hospital mortality (left) or discharge from hospital (right) for HA-COVID-19 cases and CA-COVID-19 cases. Dotted vertical lines denote the 4 periods of interest: 0–1 day, 2–8 days, 9–14 days, and 15–30 days.

Figure 1 shows the cumulative incidence plots for patient transfer to an ICU or IMCU or all-cause in-hospital mortality or discharge from the hospital for HA-COVID-19 and CA-COVID-19 cases.

**Figure 2.**
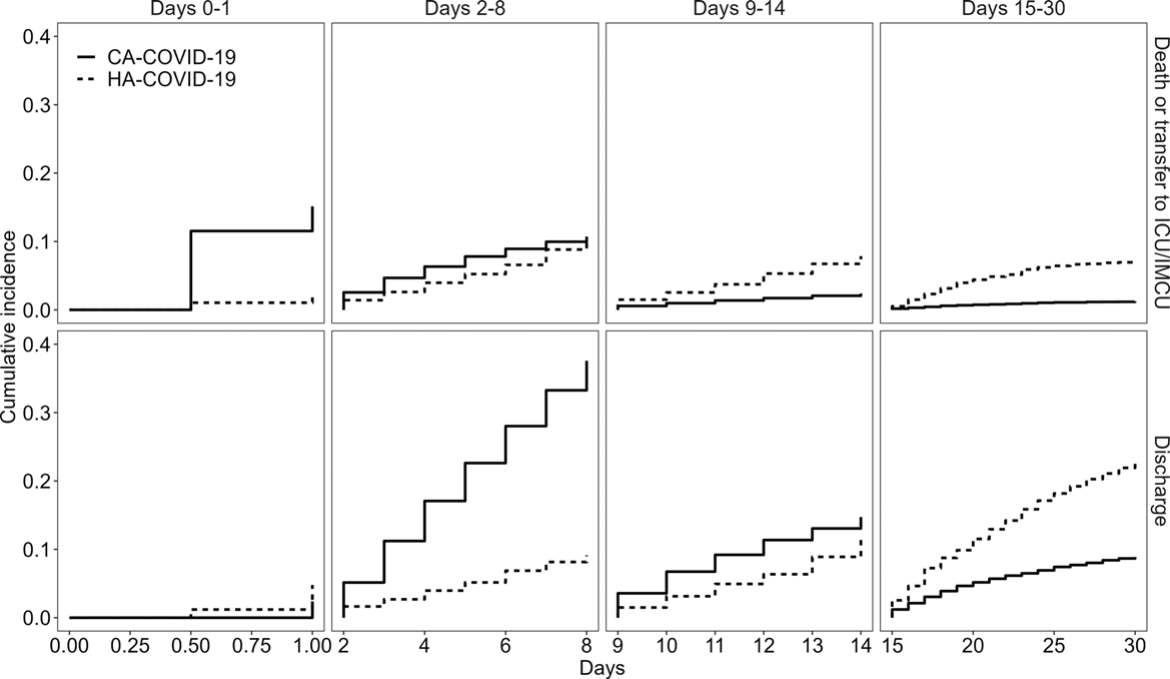
Cumulative incidence curves of patient transfer to an ICU or IMCU or all-cause in-hospital mortality (top) or discharge from hospital (bottom) for HA-COVID-19 cases and CA-COVID-19 cases over 4 periods of interest: 0–1 day, 2–8 days, 9–14 days, and 15–30 days.

To ensure proportional hazards during follow-up, time was partitioned into periods: 0–8 days and 9–30 days for patient transfer to an ICU or IMCU or in-hospital mortality; and 0–1 day, 2–14 days, and 15–30 days for patient discharge from the hospital. The csHRs and sHRs were estimated for each period for each end point of interest. By combining these periods, 4 periods of interest were defined: 0–1 day, 2–8 days, 9–14 days, 15–30 days (Figs. 1 and 2). When considering the cumulative incidence by each of these 4 periods, Figure 2 shows that the largest increase in cumulative incidence of transfer to an ICU or IMCU or all-cause in-hospital mortality among CA-COVID-19 cases occurred in the first 8 days following hospital admission.

Table 4 shows the corresponding csHRs and the sHRs for patient transfer to an ICU or IMCU or all-cause in-hospital mortality and discharge from the hospital for HA-COVID-19 cases compared to CA-COVID-19 cases over the respective periods. HA-COVID-19 patients had a lower csHR for transfer to an ICU or IMCU or all-cause in-hospital mortality during days 0–8 (csHR day 0–8, 0.43; 95% CI, 0.33–0.56) and a lower csHR for discharge (0.25; 95% CI, 0.20–0.31) from day 1 to day 8. In contrast, from day 9 onward, HA-COVID-19 cases had a higher csHR for transfer to an ICU or IMCU or all-cause in-hospital mortality (csHR day 8–30, 1.49; 95% CI, 1.20–1.85) but with no significant effect on the rate of discharge (csHR, 0.83; 95% CI, 0.61–1.14). Estimates of sHR followed a similar trend to those of csHR for each of the periods and reflects the cumulative incidence curves displayed in Figures 1 and 2. That is, HA-COVID-19 cases remained in the hospital longer than CA-COVID-19 cases, which indirectly increased their risk of transfer to an ICU or IMCU or in-hospital death over time. The results following multiple imputation of covariate data were consistent with those from the complete case analysis.

**Table 4.** Estimated Cause-Specific Hazard Ratios (csHR) and Subdistribution Hazard Ratios (sHR) for Patient Transfer to an ICU or IMCU or All-Cause In-Hospital Mortality and Discharge from Hospital for HA-COVID-19 cases and CA-COVID-19 Cases Using a Multivariate Regression Model^a^

HA-COVID-19 vs CA-COVID-19	Cause-Specific Hazard Ratio			Subdistribution Hazard Ratio		
	csHR	95% CI	*P* Value	sHR	95% CI	*P* Value
**Primary composite end point of interest: patient transfer to an ICU or IMCU or all-cause in-hospital mortality**						
HA-COVID-19 0–8 d	0.43	0.33–0.56	<.001	0.45	0.35–0.58	<.001
HA-COVID-19 9–30 d	1.49	1.20–1.85	<.001	2.84	2.10–3.83	<.001
**Competing risk of interest: discharge from hospital**						
0–1 d	2.13	1.27–3.58	.004	2.27	1.57–3.28	<.001
2–14 d	0.25	0.20–0.31	<.001	0.36	0.27–0.48	<.001
15–30 d	0.83	0.61–1.14	.258	1.42	0.90–2.23	.134

Note. ICU, intensive care unit; IMCU, intermediate care unit; CI, confidence interval; HA-COVID-19, healthcare-associated COVID-19; CA-COVID-19, community-acquired COVID-19.

a Multivariate regression model adjusted for: sex, age (categorized as <65 and ≥65 years), BMI (categorized as <30 or ≥30 kg/m²), chronic lung disease, diabetes, chronic cardiovascular disease, chronic renal disease, malignancy and having received corticosteroids treatment.

## Discussion

We conducted a large prospective cohort study of HA-COVD-19 and CA-COVID-19 cases over time during the first and second pandemic waves using different statistical methods. By performing analyses that accounted for the competing event of discharge from the hospital and multiple confounders including clinical characteristics, we found that from day 0 to day 8 of the hospital stay for COVID-19, HA-COVID-19 cases had a lower cumulative risk of ICU or IMCU transfer or in-hospital mortality compared to CA-COVID-19 cases, but with a lower rate of discharge from hospital. This finding likely reflects the more advanced disease progression in CA-COVID-19 cases at hospitalization compared to HA-COVID-19 cases due to differences in time zero between the groups. The advantage of using this approach for considering time zero (ie, the earliest date out of date of symptom onset and date of first positive SARS-CoV-2 respiratory specimen for HA-COVID-19 cases and date of hospital admission for CA-COVID-19 cases) is that immortal time bias, or the inappropriate accounting of follow-up time in each group, is minimized in both groups.^13^ Not accounting for immortal time bias would underestimate risk of adverse outcomes in the HA-COVID-19 group if the start of in-hospital follow-up predated acquisition of COVID-19, as in the CA-COVID-19 group deaths in the community without hospitalization could not be observed in the hospital admission-based database.^13^ In contrast to the results from day 0 to day 8, from day 9 onwards, HA-COVID-19 cases had higher csHR for transfer to an ICU or IMCU or all-cause in-hospital mortality, but with no significant effect on the rate of discharge. That is, during the follow-up period, more HA-COVID-19 cases remained in the hospital, which indirectly increased their risk of transfer to an ICU or IMCU or in-hospital death by 50% compared to CA-COVID-19 cases.

The estimated crude relative risk of ICU or IMCU transfer or in-hospital mortality among HA-COVID-19 cases compared to CA-COVID-19 cases was 1.15 (95% CI, 1.07–1.23) in our study. This finding is similar to a systematic review of 21 studies, including 8,251 hospital admissions across 8 countries, conducted from January 2020 to February 2021. In this meta-analysis, HA-COVID-19 cases had a 1.3-fold increased risk of mortality compared to CA-COVID-19 cases (95% CI, 1.005–1.683), with similar risk of critical care admission (RR, 0.74; 95% CI, 0.50–1.08).^7^ However, pooling risk estimates from 2 heterogeneous populations fails to account for time-dependent differences in disease progression and clinical vulnerabilities. The conventional logistic regression analysis, adjusted for confounding factors for severe COVID-19 but without accounting time varying effects, identified an overall protective effect of hospital acquisition compared to community acquisition. Our results highlight the added value of modeling time varying effects to minimize erroneous effect estimates and conclusions.

A strength of our study is the use of a combined primary outcome of patient transfer to an ICU or IMCU and/or all-cause in-hospital mortality. This is particularly relevant given that the study period covered periods early in the pandemic when ICU and IMCU capacities were stretched and elderly patients, or patients with comorbidities, may not have always been eligible for ICU or IMCU admission when beds were limited. Indeed, when considering the primary end points separately, there was a greater proportion of deaths (18.3% vs 7.7%) and a lower proportion of ICU and IMCU transfers (8.8% vs 21.8%) among HA-COVID-19 cases compared to CA-COVID-19 cases.

More broadly, our results indicate both the clinical importance of COVID-19, with mortality rates that have been shown to be substantially higher than other respiratory viruses, such as seasonal influenza, in a nonimmune population.^14^ The proportion of patient transfers to an ICU or IMCU or all-cause in-hospital mortality among HA-COVID-19 further emphasizes the need for appropriate infection control measures to limit healthcare-related transmission.^15^

This study had several limitations. There was possible heterogeneity in detection of HA-COVID-19 cases across the 16 participating hospitals due to variations in type of healthcare facility, availability of SARS-CoV-2 testing capacities and differences in testing strategies (ie, screening strategies were not uniform across hospitals in the event of in-hospital clusters, particularly during the initial wave in 2020) and in infection control measures. These sources of heterogeneity mean that some milder HA-COVID-19 cases may have been missed, leading to a possible overestimation of severity among HA-COVID-19 cases. Secondly, we used a rather conservative definition of HA-COVID-19 by which cases were considered healthcare acquired if infection occurred >5 days after hospital admission. At the time of this study, there was no consensus on the definition of HA-COVID-19.^14^ To reflect the median incubation period for SARS-CoV-2, we used 5 days after hospital admission. In 2020, other definitions in use included up to >14 days after hospital admission.^14^ The implication for our study may have been a misclassification of a proportion of CA-COVID-19 as HA-COVID-19 cases. Thirdly, our study covered a period that predated the introduction of effective COVID-19 vaccines, and although this may avoid the confounding introduced by the protective effect afforded by COVID-19 vaccines, our results may not necessarily apply to cases of COVID-19 in settings of high levels of infection- and vaccine-derived immunity. Finally, while differences in time zero between the 2 groups minimize immortal time bias, they nonetheless reflect differences in disease progression, as described above.

In conclusion, in this large, prospective, cohort study of RT-PCR-confirmed COVID-19 inpatients across 16 hospitals in Switzerland, HA-COVID-19 patients remained in the hospital longer and had an increased risk of transfer to an ICU or IMCU or in-hospital death over time compared to CA-COVID-19 patients. Our time-varying analysis shows how this risk varies over time and emphasizes the importance of appropriate epidemiologic methods and statistical analyses to inform clinicians and policy makers about the true risk of clinical outcomes related to HA-COVID-19.
